# Oxidative stress: therapeutic approaches for cervical cancer treatment

**DOI:** 10.6061/clinics/2018/e548s

**Published:** 2018-11-27

**Authors:** Gabriela Ávila Fernandes Silva, Rafaella Almeida Lima Nunes, Mirian Galliote Morale, Enrique Boccardo, Francisco Aguayo, Lara Termini

**Affiliations:** I Instituto do Cancer do Estado de Sao Paulo ICESP, Centro de Investigacao Translacional em Oncologia, Hospital das Clinicas HCFMUSP, Faculdade de Medicina, Universidade de Sao Paulo, Sao Paulo, SP, BR; II Departamento de Radiologia e Oncologia, Faculdade de Medicina FMUSP, Universidade de Sao Paulo, Sao Paulo, BR; III Laboratorio de Oncovirologia, Departamento de Microbiologia, Instituto de Ciencias Biomedicas, Universidade de Sao Paulo, Sao Paulo, SP, BR; IV Centro Avanzado de Enfermedades Cronicas (ACCDiS), Facultad de Medicina, Universidad de Chile, Santiago, Chile; V Departamento de Oncologia Basico Clinica, Facultad de Medicina, Universidad de Chile, Santiago, Chile

**Keywords:** Tumor Markers, Human Papillomavirus, Redox System, Cervical Cancer

## Abstract

**OBJECTIVES:** Oxidative stress results from an imbalance between the generation and elimination of oxidant species. This condition may result in DNA, RNA and protein damage, leading to the accumulation of genetic alterations that can favor malignant transformation. Persistent infection with high-risk human papillomavirus types is associated with inflammatory responses and reactive oxygen species production. In this context, oxidative stress, chronic inflammation and high-risk human papillomavirus can act in a synergistic manner. To counteract the harmful effects of oxidant species, protective molecules, known as antioxidant defenses, are produced by cells to maintain redox homeostasis. In recent years, the use of natural antioxidants as therapeutic strategies for cancer treatment has attracted the attention of the scientific community. This review discusses specific molecules and mechanisms that can act against or together with oxidative stress, presenting alternatives for cervical cancer prevention and treatment.

## INTRODUCTION

### Oxidative stress: general aspects

Oxidative stress (OS) results from an imbalance in the formation and elimination of oxidant species. Accumulation of these molecules may lead to cell dysfunction as a consequence of accumulated oxidative modifications in several biomolecules 1. Free radical generation represents a continuous physiological process that results from biological functions, including metabolism and inflammation. In the mitochondria, cytochrome P450 and peroxisomes are the major endogenous factors leading to reactive oxygen species (ROS) and reactive nitrogen species (RNS) formation. Other exogenous factors, such as radiation, tobacco smoking, chemotherapy and diet, are also important inductors of free radical production 2. Intermediate reactive species that are naturally produced under physiological conditions have a crucial role in metabolic regulation, the cell cycle and intracellular signaling pathways 3.

To maintain redox homeostasis, protective molecules called “antioxidant defenses” act to preserve the balance between formation and removal of ROS and RNS. Cellular antioxidant systems are currently divided into enzymatic and non-enzymatic groups. The enzymatic group comprises catalase, superoxide dismutase (SOD), glutathione peroxidase (Gpx) and glutathione-S-transferase (GST). The non-enzymatic group is composed of molecules such as vitamins C and E, lipoic acid, carotenoids, flavonoids and others 4.

**Figure 1:**
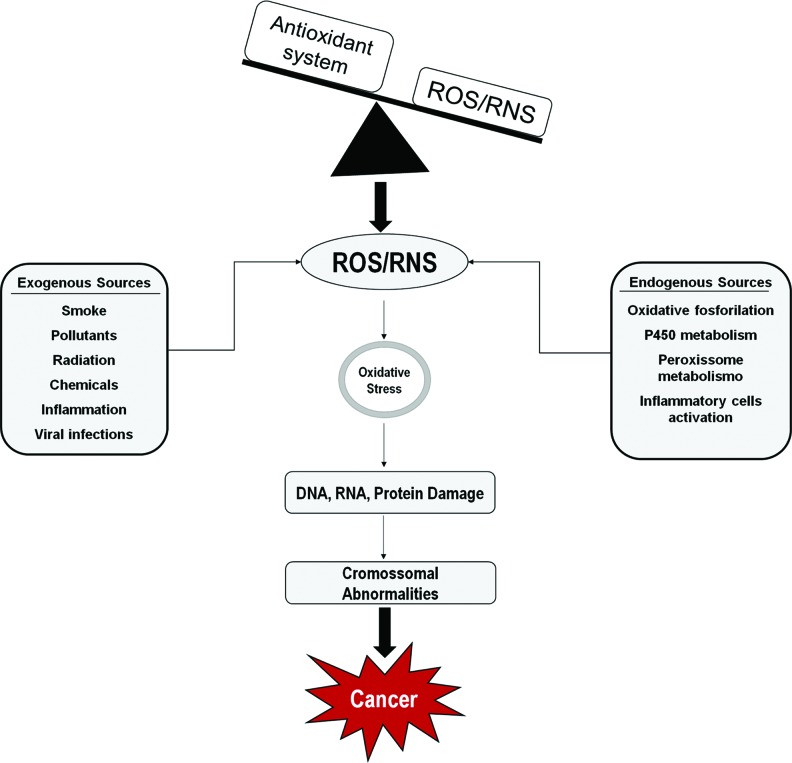
Several factors, such as environmental pollutants, chemicals, tobacco smoking, chronic inflammation, among others, generate reactive oxygen and nitrogen species (ROS/RNS). An imbalance between oxidant species and the antioxidant system results in DNA, RNA and protein damage, which may lead to the accumulation of genetic alterations and promote malignant transformation. Sources of ROS/RNS.

Excess levels of unneutralized free radicals and cellular active intermediates are the major cause of OS 5-7. The presence of high concentrations of oxidized biomolecules is associated with alterations in aerobic metabolism, inflammatory response, exposure to UV radiation, hypoxia, anomalous cell proliferation and viral infections, among others. Therefore, OS is directly associated with several pathological conditions including tumors associated with human papillomavirus (HPV) infection (Figure 1) 8-10.

### Human papillomavirus infection and cancer

Persistent infection with high-risk HPV types (HR-HPV) is the main etiological cause for the development of several epithelial tumors at different anatomic locations. The most strongly HPV-associated malignancy is cervical carcinoma, where almost all tumors are positive for HR-HPV DNA 11. Cervical carcinoma is the second most common type of cancer in women worldwide, with approximately 530,000 new cases and 270,000 deaths per year, of which more than 85% occur in developing countries. HPV infection is implicated in a variable, although consistently high, proportion of vaginal, vulvar, anal, penile and head and neck carcinomas 12,13.

In addition to HPV infection, several cofactors contribute to cervical cancer development. These include low socioeconomic status, early sexual initiation, multiple sexual partners, smoking, multiparity, immunosuppression and use of oral contraceptives 14,15. Cervical cancer is mostly a consequence of the continuous evolution of non-invasive precursor lesions called cervical intraepithelial neoplasia (CIN) that are characterized by different degrees of cellular atypia (dysplasia) 16. CIN is divided into the following groups: CIN 1, characterized by mild dysplasia; CIN 2, which represents moderate dysplasia; and CIN 3 or carcinoma in situ, characterized by a severe dysplasia that may progress to an invasive squamous cell carcinoma (SCC). Moreover, changes in the glandular epithelium of the cervix, caused by HPV and other cofactors, are associated with the development of cervical adenocarcinoma 16,17. Furthermore, in persistent viral infection, HPV-induced carcinogenesis involves genetic and epigenetic changes that affect the expression of different cellular proto-oncogenes and tumor suppressor genes. Usually, this process requires an extensive period to accumulate sufficient alterations to trigger and sustain tumor development 18,19. Therefore, immune system evasion and HPV persistence are crucial factors for tumorigenesis 20. This is highlighted by the fact that the great majority of HPV infections are self-limited and spontaneously resolved in few months, and cancer development affects only a small proportion of infected individuals.

E6 and E7 viral oncoproteins have a central role in tumor development. In high-grade lesions and tumors, these proteins are upregulated mainly due to the loss or disruption of the viral E2 gene (see below), which normally restricts the expression of viral oncogenes 21. The HPV oncoproteins E6 and E7 interact with several tumor suppressor factors and cell cycle regulators, such as p53 and pRB, respectively. These interactions lead to their deregulation and cell immortalization. In addition, E6 and E7 affect many other cellular proteins with several regulatory activities, promoting immune evasion, resistance to apoptosis and epithelial cell proliferation 22. The pleiotropic functions of these oncoproteins deserve to be noted, since they result from a well-adapted evolutionary relationship between virus and host that is essential for HPV tumorigenesis 23. Persistent viral infection, abnormal metabolism of keratinocytes expressing HPV oncogenes and non-effective chronic inflammatory responses lead to ROS production 24 and cause OS, contributing to the cell transformation process 25.

Alterations in expression and activity of some antioxidant proteins, including peroxiredoxins, catalase, quinone oxidoreductase-1 and superoxide dismutase (SOD) family proteins, can be detected in pre-neoplastic and neoplastic tissues associated with HPV infections. For example, expression of SOD2, a crucial antioxidant enzyme responsible for controlling the redox status of normal and tumor cells, is upregulated in several HPV-associated tumors, including penile and cervical carcinomas 26-29. Furthermore, results from different studies have established that the HR-HPV E6 and E7 oncoproteins can modulate OS to favor the accumulation of mutations, a fact that is directly related to cell transformation 23,30,31.

### Oxidative Stress and inflammasomes

Inflammasomes are cytosolic multiprotein complexes that assemble after exposure to pathogens or danger-associated molecular patterns, leading to caspase activation and, consequently, secretion of inflammatory cytokines and cell death. Although different inflammasome complexes have been described, with unique activation triggers, they typically consist of a cytosolic pattern recognition receptor (PRR) such as RIG-I-like receptor (RLR), AIM2-like receptor (ALR) or nucleotide-binding domain and leucine-rich repeat-containing (NLR) protein, an adaptor protein (ASC) and pro-caspase-1. The OS generated during HPV infections can be modulated by the infected keratinocytes as well as by activated neutrophils and macrophages 32-34. Moreover, ROS regulate inflammasomes in response to oncogenic viral infections, such as HR-HPV 35-37. Inflammasomes are important because they activate caspase-1 in response to invading pathogens. Therefore, caspase-1 cooperates with apoptosis-associated speck-like protein containing a C-terminal caspase-recruitment domain (ACS), processing the proinflammatory cytokines interleukin 1 beta (IL-1β) and interleukin 18 (IL-18) into their mature forms 38.

Furthermore, protein absent in melanoma-2 (AIM2) activates caspase-1 and inflammasomes by binding to foreign cytoplasmic double stranded DNA (dsDNA) and its adaptor ACS 39-41. Interestingly, HPV16 activates AIM2 inflammasomes and promotes upregulation of proinflammatory cytokines such as IL-1β, IL-1α, and IL-18, which are increased in lesions that evolve to cervical cancer 36.

### Oxidative Stress and Nuclear Factor-kappa B

Nuclear factor-kappa B (NF-κB) is a pleiotropic transcription factor composed of dimers of five different members of the Rel transcription factor family that include p105/p50, p100/p52, RelA, RelB and cRel 42. This factor is found in the cytoplasm from the majority of resting cells, and after activation by a wide range of signals, such as viral infections, bacterial lipopolysaccharides (LPS), DNA damage, OS and proinflammatory cytokines, this protein translocates to the nucleus and regulates the expression of hundreds of genes 42-44. Genes encoding cytokines and chemokines, growth factors, cell adhesion molecules, anti-apoptotic proteins and antioxidant enzymes are regulated by NF-κB 45,46.

NF-κB plays a central role in inflammation and immune responses but also acts in different cellular processes, including development, cell growth, survival and proliferation. Furthermore, this transcription factor has a role in many pathological conditions, including cancer, where it may be involved in tumor growth and metastasis 42,44.

ROS levels can be affected by NF-κB activity through its influence on the expression of antioxidant proteins, including SOD1, SOD2, Ferritin Heavy Chain (FHC), Glutathione S-transferase pi (GST-pi), Glutathione peroxidase-1 (Gpx1) and Dihydrodiol dehydrogenase (DDH1). However, ROS may activate or inhibit NF-κB signaling depending on the signaling pathway step (upstream or downstream) and cell type analyzed 44,. A recent study showed that Pirin, another NF-κB regulator and OS sensor, is overexpressed in cervical cancer-derived cell lines and in oral mucosal keratinocytes expressing HPV16 oncogenes 50. This observation shows another mechanism for HR-HPV-dependent NF-κB activation in cervical cancer cells and further supports the potential use of this factor as a therapeutic target.

NF-κB activation by free radicals or enzymes is a complex event and can cause various biological effects. For example, its activation by SOD2 has been associated with lung adenocarcinoma progression and poor prognosis 51. Additionally, OS generated in tumors can impair NF-κB translocation in thymic T cells, which become vulnerable to tumor necrosis factor alpha (TNFα)-mediated apoptosis.

Interestingly, curcumin, a natural antioxidant that is discussed below, could prevent tumor-induced thymic atrophy, restoring NF-κB activity, and thus acted as an immunorestorative compound 43.

### Oxidative Stress and DNA damage

A crucial event in HPV-mediated malignant transformation is the integration of HR-HPV DNA into the cell genome. This event results in E6 and E7 oncogene overexpression, which leads to the destabilization of p53 and pRb tumor suppressor proteins, respectively 52. HR-HPV integration is facilitated by generation of DNA damage/double strand breaks (DSBs) 53. Notably, DNA damage causes DNA oxidation, leading to a plethora of genome alterations, including deletions, insertions, point mutations, DSBs and translocations 54,55.

Genome oxidative damage was previously shown to be strongly involved in epigenetic alterations 56. In an elevated oxidative DNA damage environment, the production of 8-oxo-2′-deoxyguanosine (major product of DNA oxidation) coincides with increased HPV infection, viral-host integration and cervical dysplasia 57. Furthermore, HR-HPV integration into the cell genome generally causes the disruption of the HPV E2 ORF, resulting in E2 gene inactivation and upregulation of E6 and E7 transcription 53.

DNA repair pathways can be affected by HPV oncoproteins, resulting in the inactivation of important groups of genes, such as base excision repair (BER), nucleotide excision repair (NER), DNA mismatch repair (MMR), microhomology-mediated end joining (MMEJ), Fanconi anemia (FA), ataxia-telangiectasia mutated (ATM), and the ATM and Rad3-related (ATR) genes 58. These pathways are implicated in HR-HPV-mediated cervical cancer 59,60.

During HR-HPV infection, apoptosis is hindered by disruption of many regulatory pathways, which results in altered cell proliferation associated with accumulation of mutations and alterations in gene expression 24. In HPV16-related invasive cervical cancer, E2 binding sites (E2BSs) at the viral long control region (LCR) are epigenetically regulated by hypermethylation, dampening E2 protein binding and resulting in the upregulation of E6 and E7 expression 61-63. Moreover, DNA methylation functions as a barrier protecting the virus from immune surveillance 64,65. In summary, theoretical considerations suggest that OS, chronic inflammation, epigenetic alterations and HR-HPV can act in a synergistic manner during oncogenesis.

## THERAPEUTIC APPROACHES

### Oxidative stress control: natural antioxidants

Recently, natural compounds with chemopreventive and chemotherapeutic properties, which have antioxidant features, have received increased attention 66. An inverse association was observed between antioxidant factors present in the diet and HPV tumorigenesis, suggesting that natural antioxidants may protect against HPV persistence and tumor development 67,68. Furthermore, studies using natural compounds present in plant extracts, such as curcumin and resveratrol, have been shown to sensitize tumor cells to radio- and chemotherapy. These observations indicate that natural antioxidants have the potential to reduce the establishment and progression of precursor lesions. Molecules with these properties may be used as a complementary approach for cancer treatment 69,70.

### Curcumin

**Figure 2:**
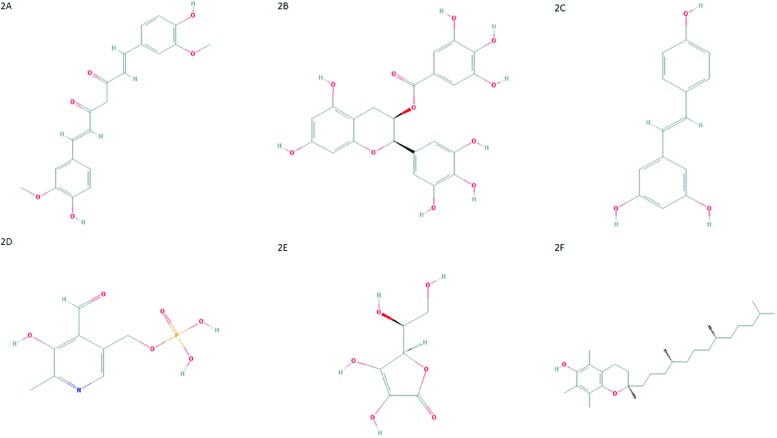
**(A)** Chemical structure of curcumin. PubChem CID 969516; **(B)** Chemical structure of epigallocatechin-3-gallate. PubChem CID 65064; **(C)** Chemical structure of resveratrol. PubChem CID 445154; **(D)** Chemical structure of pyridoxal-5′-phosphate. PubChem CID 1051; **(E)** Chemical structure of ascorbic acid. PubChem CID 54670067; **(F)** Chemical structure of α-tocopherol. PubChem CID 14985.

Curcumin, also known as diferuloylmethane (Figure 2A), is one of the main compounds present in turmeric (*Curcuma longa - Zingiberaceae family*) rhizome 71. Moreover, curcumin scavenges ROS and RNS due the activity of the OH or CH2 group of the β-diketone moiety. In other words, curcumin reacts with free radicals by electron transference followed by proton loss or direct H-atom abstraction, mainly due to the phenolic OH group 72-74. The value of curcumin has been widely addressed and discussed in scientific literature. This compound has anti-inflammatory, antitumoral, antioxidant, and cardioprotective activities and several other properties 75-82. Curcumin can inhibit NF-κB activation through restriction of IκBα kinase and Akt activation 83-89. This change results in the inhibition of NF-κB-regulated gene products that control apoptosis, proliferation, invasion and angiogenesis 90,91.

Moreover, curcumin downregulates other crucial transcription factors responsible for controlling cell growth and survival pathways, including signal transducer and activator of transcription 3 (STAT3), cyclooxygenase 2 (COX2), Akt, antiapoptotic proteins and activator protein 1 (AP1) 69,92,93. In addition, this compound can downregulate angiogenesis *in vivo*, inhibiting the activity of proangiogenic molecules, such as fibroblast growth factor 2 (FGF-2), matrix metalloproteases and COX2, as noted previously 94,95. Finally, curcumin can induce the expression of antioxidant enzymes through nuclear factor erythroid 2–related factor 2 (NRF2) activation 57.

Due to its biological properties and low toxicity, curcumin is an interesting alternative to therapeutic agents used for the treatment of several types of cancer 96-98. Various studies have addressed the antitumor effects of curcumin in experimental models of cervical cancer. For instance, the HPV16-positive cervical cancer-derived cell line SiHa could be sensitized to cisplatin treatment by curcumin co-administration. This treatment inhibited NF-κB activity and increased cell death 99. Treatment with curcumin also sensitized cervical cancer-derived cells and HPV-positive tumors to taxol and paclitaxel, respectively. Again, these effects involve the downregulation of survival signals regulated by NF-κB and Akt, such as phosphorylation of MAPKs and induction of Cyclin-D1, COX2, X-linked inhibitor of apoptosis protein (XIAP) and cellular inhibitor of apoptosis protein-1 (cIAP1) 100-102.

In addition, curcumin was shown to downregulate HPV18 transcription in HeLa cells, inhibiting the AP1 pathway and reversing the expression patterns of the c-fos and fra-1 transcription factors 103. Furthermore, depending on the concentration and duration of treatment with curcumin, a cytotoxic effect could be observed in cervical cancer cells 104. Interestingly, another study showed that thioredoxin reductase plays a role in the radiosensitizing effect of curcumin on cervical cancer cells 105. Due to its chemopreventive activity in several types of cancer, curcumin has been evaluated in preclinical studies and clinical trials 106. A study was developed using curcumin in a specific formulation (Meriva) to investigate its impact on the quality of life of 160 cancer patients. Study participants presented solid or hematological malignancies and were undergoing radio- or chemotherapy following surgical treatment, with significant side effects and increased oxidative stress. Despite the study limitations, which were recognized by the authors, the results provided the first clinical evidence that curcumin may reduce the side effects of cancer treatment and improve patient quality of life. Notably, curcumin has extremely low oral bioavailability, and that it is necessary to develop formulations that allow its clinical use 107.

### Epigallocatechin-3-gallate (ECGC)

Another important polyphenol with anticancer properties is epigallocatechin-3-gallate (ECGC). This compound (Figure 2B) it is the major catechin from Camellia sinensis, found in green tea 108. This potent antioxidant scavenges ROS and has antiproliferative, antiangiogenic, antimetastatic and proapoptotic effects in several tumor models 109-119.

EGCG is a potent inhibitor of the Akt/NF-κB and mammalian target of rapamycin (mTOR) signaling pathways inducing apoptosis and cell survival mechanisms. Moreover, EGCG can cause NRF2-mediated antioxidant induction and reduce inflammation 120. Notably, the activation of NRF2 is a key target of cytoprotective agents, and its upregulation results in increased expression of Heme oxygenase-1 (HO-1 antioxidant enzyme) 121. Furthermore, NRF2 prevention of inflammation by ROS occurs though the inactivation of NF-κB 122. The ECGC antiproliferative effect is related to the accumulation of cells in the G1 phase of the cell cycle, followed by apoptosis 123. Interestingly, this compound can also induce apoptosis in cervical cells extracted from fresh tissues 124.

A recent study showed that EGCG exhibits free radical scavenging properties in cells isolated from cervical cancer biopsies, decreasing cell proliferation and increasing the activity of antioxidant enzymes, such as SOD and Gpx 125. In addition to targeting cell growth and apoptosis, this compound can further inhibit telomerase action, impairing initiation and development of cervical lesions 126,127. As mentioned before, ECGC also displays antiangiogenic features through inhibition of hypoxia-inducible factor 1 alpha (HIF-1α) and of vascular endothelial growth factor (VEGF), as a result of proteasome activity and blockage of PI3K/Akt and ERK1/2-regulated pathways 128.

Interestingly, EGCG can act synergistically with cisplatin, inhibiting the growth of cervical cancer-derived cell lines. This finding suggests that cisplatin treatment is potentiated with EGCG in HeLa cells, regulating pathways involved in cell survival and apoptosis. This combination may improve cervical cancer treatment, especially in the context of chemoresistance to cisplatin 129.

A clinical trial was carried out to verify the efficacy of two green tea extract-derived compounds, EGCG and polyphenon E, in a study involving ninety women with HPV-related cervical lesions. These patients were divided in four different groups, according to the following regimens: local application of a polyphenon E ointment, daily oral dose (200 mg) of polyphenon E, both local and oral polyphenon E and 200 mg daily oral dose of EGCG. Patients treated with EGCG or polyphenon E responded positively to the treatment, with a reduction in HPV DNA copies and regression of virus-associated lesions, while the majority of the untreated group showed no improvement 130.

### Resveratrol

Resveratrol or 3,5,4-trihydroxystilbene (Figure 2C), a phytoalexin found in grapes, peanuts, blueberries and other food products, shows antitumor effects by interacting with several molecules involved in tumor development, such as first apoptosis signal receptor (Fas), MAPK, NF-κB and AP1 131,132.

Resveratrol exhibits antiproliferative effects on cervical cancer-derived cell lines. This effect was characterized by accumulation of cells in the S-phase of the cell cycle 70. The result was further confirmed by another study, using a double dosage of resveratrol, which induced a transient and reversible accumulation of HeLa cells in the S-phase 133. Recently, the glycoprotein cyclooxygenase pathway was shown to be a crucial target of resveratrol. However, it apparently does not directly regulate COX expression. Instead, it may regulate other enzymes related to prostaglandin synthesis acting downstream of the COX pathway 134.

The chemopreventive efficacy of resveratrol has already been demonstrated in hepatocellular, skin, prostate and lung cancers, through several regulatory pathways 135,136. Nevertheless, its exact mechanism in cervical cancer remains to be investigated, which limits its therapeutic applications. Recent data demonstrated that resveratrol inhibits HeLa cell growth and induces apoptosis in a dose- and time-dependent manner. In addition, these cells presented apoptotic characteristics, such as cell shrinkage, formation of apoptotic bodies and DNA fragmentation 137. Interestingly, resveratrol can reduce the active form of Akt, leading to autophagy, cell cycle arrest and apoptosis of SiHa and HeLa cells. Moreover, it can destabilize lysosomes, resulting in cathepsin L (cat L) translocation to the cytosol 131,134,138,139. When the cat L enzymatic activity increases in the cytosol, cytochrome c is released from the mitochondria, leading to cell death by apoptosis. Triggering apoptosis through cat L causes lysosome membrane permeabilization and release of its proteases to the cytosol. 138. Studies conducted by Rezk et al. 140 showed that resveratrol improves the effectiveness of cisplatin and doxorubicin chemotherapy, suggesting it can be used in cervical cancer treatment.

### Pyridoxal-5′-phosphate (Vitamin B6)

Pyridoxal-5′-phosphate (PLP) is the bioactive form of vitamin B6 (Figure 2D). There are three non-phosphorylated vitamin B6 precursors, catalyzed by pyridoxal-kinase (PDXK) into their phosphorylated forms, including PLP 141.

Vitamin B6 is required as a co-enzyme for many biochemical reactions, including amino acid synthesis and catabolism, bioactive amine synthesis (histamine, serotonin, dopamine), hemoglobin synthesis, and glycogenolysis 142-144. Its antioxidant properties include direct effects, due to reaction of its hydroxyl and amine groups with peroxy radicals, or indirect effects, due to its role in homocysteine to cysteine conversion. Cysteine is a necessary substrate for glutathione synthesis and is essential for an ideal redox balance 145.

Vitamin B6 could sensitize several cancer cell lines to apoptosis after cisplatin-mediated DNA damage by depleting intracellular glutathione. Furthermore, low PDXK expression was associated with poor prognosis in two independent cohorts of non-small cell lung cancer patients 146.

In a cohort of thirty-three hepatocellular carcinoma patients that randomly received placebo or vitamin B6 (50 mg/d), the results indicated that vitamin B6 improved the antioxidant capacity by reducing homocysteine levels in patient plasma 145.

### Ascorbic acid (vitamin C)

Recent data have shown that vitamin C, also known as ascorbic acid (Figure 2E), is another antioxidant molecule and can increase the effect of chemotherapeutic agents without augmenting their toxicity in normal cells.

In SiHa cells, the association of vitamin C and cisplatin enhanced cisplatin-mediated apoptosis induction through a *p53*-mediated pathway. Therefore, low concentrations of cisplatin were required to induce cancer cell death. Hence, it is tempting to speculate that, in combination with vitamin C, low amounts of cisplatin could be used in cancer patients, reducing its side effects 147.

Interestingly, ascorbyl stearate (ASC-S), a fatty acid ester derivative from ascorbic acid, exhibits a potent proapoptotic activity. Recently, Mane et al. 148 described the proapoptotic effect of this molecule on HeLa cells due to the induction of changes in the mitochondrial membrane permeability, cytochrome c release and caspase-3 and NF-κB activation.

Finally, the direct impact of ascorbic acid on cervical carcinogenesis has been suggested. A cross-sectional study published by Hwang et al. 149 showed that a dietary supplementation including vitamin C might reduce the risk of CIN in women with HR-HPV infection.

### α-Tocopherol (vitamin E)

Results from previous studies suggest the existence of an association between plasma levels of vitamin E, also known as α-tocopherol or 5,7,8-trimethyltocol (Figure 2F), and HPV infection status. For example, women with CIN were shown to exhibit decreased plasma levels of this antioxidant compound, which may reflect the existence of elevated OS 150,151. Similar results showed that patients with cervical cancer had low levels of vitamin E 152. Additionally, Hu and co-workers 153 suggested that vitamin E intake or high circulating levels may reduce the risk of CIN and cervical cancer. Furthermore, a study published by Palan et al. 154 supports previous data suggesting that α-tocopherol and other antioxidants have an important protective role *in vivo*. In addition, the maintenance and regeneration of α-tocopheryl quinone (a non-oxidized form of α-tocopherol) may also have an important role in CIN and in cervical cancer. However, further investigations of α-tocopheryl quinone as a potential marker of OS in precancerous lesions and cervical cancer are strongly needed 154.

### Oxidative stress induction: traditional chemotherapy

Despite the positive results with the use of the antioxidant compounds described above, traditional cancer chemotherapy still relies on the induction of OS triggered by ROS levels higher the than tumor elimination capacity, which contributes to tumor cell death 155. Nevertheless, evidence has shown that upregulation of antioxidant enzymes by cancer cells has a relevant role in OS control and possibly in drug resistance 156.

One of the most important systems involved in cell protection against elevated levels of free radicals, including those induced by chemotherapy and radiotherapy, is composed of glutathione (GSH) and GSH-related enzymes 157. Regarding this issue, a study analyzed the role of the GSH redox system in drug-resistant glioblastoma multiforme cells. Temozolomide (TMZ)-resistant glioma cells showed low levels of ROS and high GSH and glutathione reductase levels, exhibiting greater antioxidant capacity than sensitive cells, suggesting that this redox system may be an important therapeutic target 158. Interestingly, HPV proteins can also modulate ROS by regulating GSH and SOD levels 159. In fact, a relationship between GSH levels in cervical cancer cells and resistance to doxorubicin treatment, an anticancer drug that induces DNA damage and ROS production, was observed 160.

An alternative treatment for cervical cancer involves molecules capable of inhibiting antioxidant pathways. For example, exposure of HeLa cells to pinostrobin, a dietary bioflavonoid, was associated to reduced cell viability and downregulated GSH and NO2- levels. Pinostrobin-associated cell death involves upregulation of apoptotic extrinsic and intrinsic pathways components, as well as DNA and mitochondrial damage, probably as a consequence of ROS accumulation 161.

Another compound, auranofin, an inhibitor of thioredoxin reductase, can also trigger apoptosis while depleting GSH and increasing ROS intracellular levels. In addition, its effect on cervical cancer cell lines can be intensified in combination with a GSH synthesis inhibitor, L-buthionine sulfoximine 162. By inhibiting antioxidant effects, these treatments overcome HPV resistance to ROS induction, leading to cell death. Finally, therapeutic approaches using ROS inductors or antioxidant molecules such as SOD and SOD-mimetics combined with radiotherapy or chemotherapy have been investigated and may constitute viable alternatives for cancer treatment in the future 163.

### Enzyme inhibitors

The use of enzyme inhibitors such as tyrosine kinase inhibitors (TKIs) to modulate ROS effects constitutes an important alternative to cancer therapies currently under investigation. Sunitinib, for instance, is the most common TKI administered in clinical medicine, and it effectively blocks VEGF receptors 164-166, platelet-derived growth factor receptor (PDGFR) *alfa* and *beta*, and c-Kit 167.

According to some published data, sunitinib could have antioxidant activity due to the improved lipid peroxidation and increased GSH levels observed after cisplatin treatment, reducing OS-triggered side effects and improving chemotherapeutic efficacy 168. When combined with chloroquine, sunitinib augments NOS production, causing an accumulation of RNS and triggering apoptosis; it also increases the levels of GSH, which in turn can interrupt apoptosis 169. In addition, a combination of chloroquine and sunitinib induces increased toxicity in breast, cervical, colorectal, hepatocellular, laryngeal and prostate cancer cell lines as well as in mouse tumor models 169. However, based on the current knowledge, the potential impact of these effects on patients' outcome are still unclear.

Another approach involving enzyme inhibitors was applied in a study that developed a new radiosensitization technique using a hydrogen peroxide solution (Oxydol) named KORTUC (Kochi Oxydol-Radiation Therapy for Unresectable Carcinomas). This approach aimed to treat patients with local advanced unresectable neoplasms, through tumor antioxidative enzyme blockage, leading to tumor sensitization to radiotherapy 170. The same group published further clinical studies showing that this method may be a safe, well-tolerated, and efficient approach 170-173. However, it is also a broad field of study.

Oxidative stress is a consequence of an imbalance between pro- and antioxidant factors. The antioxidant system maintains the oxidative process within physiological limits, preventing injuries that can culminate in irreparable systemic damage and diseases.

Signaling pathways related to redox control in HPV-mediated carcinogenesis represent promising targets for cancer treatment. Some antioxidant molecules, including natural compounds, may be useful preventive or therapeutic alternatives. Despite the positive results of antioxidant compounds, OS induction is still an effective therapeutic approach used in traditional chemotherapy. This approach aims to enhance reactive species production to levels that overcome the tumor elimination capacity, leading to tumor cell death. However, evidence has shown that an increase in antioxidant enzymes by cancer cells has an important role in OS control and possibly in drug resistance.

Current knowledge indicates that several molecules and enzyme inhibitors act to modulate ROS and that they are some of the most significant therapeutic targets for anticancer compound development and should be extensively studied. Despite several lines of evidence based on *in vitro* or animal model studies, relatively few clinical trials have been carried out. These trials are essential for the establishment of new therapies targeting oxidative stress, including those using natural compounds.

## AUTHOR CONTRIBUTIONS

Silva GA contributed to the abstract, introduction, figures, therapeutic approaches and conclusion and revised the text and references. Nunes RA contributed to preparation and revision of the text. Morale MG contributed to the introduction and revised the text and references. Aguayo F and Boccardo E revised the text. Termini L prepared and revised the text.
